# Data on eye behavior during idea generation and letter-by-letter reading

**DOI:** 10.1016/j.dib.2017.09.009

**Published:** 2017-09-12

**Authors:** Sonja Walcher, Christof Körner, Mathias Benedek

**Affiliations:** Institute of Psychology, University of Graz, Universitätsplatz 2, 8010 Graz, Austria

## Abstract

This article includes the description of data information from an idea generation task (alternate uses task, (Guilford, 1967) [1]) and a letter-by-letter reading task under two background brightness conditions with healthy adults as well as a baseline measurement and questionnaire data (SIPI (Huba et al., 1981) [2]; DDFS (Singer and Antrobus, 1972) [3], 1963; RIBS (Runco et al., 2001) [4]). Data are hosted at the Open Science Framework (OSF): https://osf.io/fh66g/ (Walcher et al., 2017) [5]. There you will find eye tracking data, task performance data, questionnaires data, analyses scripts (in R, R Core Team, 2017 [6]), eye tracking paradigms (in the Experiment Builder (SR Research Ltd., [7]) and graphs on pupil and angle of eye vergence dynamics. Data are interpreted and discussed in the article ‘Looking for ideas: Eye behavior during goal-directed internally focused cognition’ (Walcher et al., 2017) [8].

**Specifications Table**

**Table:** 

Subject area	Psychology
More specific subject area	Cognitive Psychology, Eye Tracking
Type of data	Tables, text files, graphs, code
How data was acquired	Eye tracking: EyeLink1000Plus, surveys
Data format	Raw, processed, analyzed
Experimental factors	Participants (*N*=50) performed two tasks (letter-by-letter reading task and idea generation task) under two background brightness conditions (bright: rgb(204,204,204), dark (102,102,102)). One practice and eight 60 second trials were presented per task, half under bright half under dark background condition. During the idea generation task, a non-sense letter-by-letter string was presented. Task performance was assessed after each trial. Additionally, a baseline measurement was obtained where participants were asked to fixate a fixation cross for 60 seconds.

Experimental features	Eye tracking data were obtained during letter-by-letter reading, idea generation and a baseline condition.
Data source location	Department of Psychology, University of Graz, Graz, Austria
Data accessibility	Data are hosted at the Open Science Framework (OSF): https://osf.io/fh66g/[5]

**Value of the data**

• Raw eye tracking data of 50 participants useful for reanalysis by other scholars.
• Useful for meta-analyses on effects of internally-directed cognition on wide range of oculometric parameters.
• Top two ideas of 50 participants on nine 60 s alternate uses trials useful for evaluation of task and future application.
• Data on mind wandering and imaginal processes related questionnaires useful for analyses of reliability and associations between them and with eye behavior.
• R-script for the calculation of angle of eye vergence from gaze position data is provided and can be used by other scholars.

## 1 Data

**Fig. 1:**
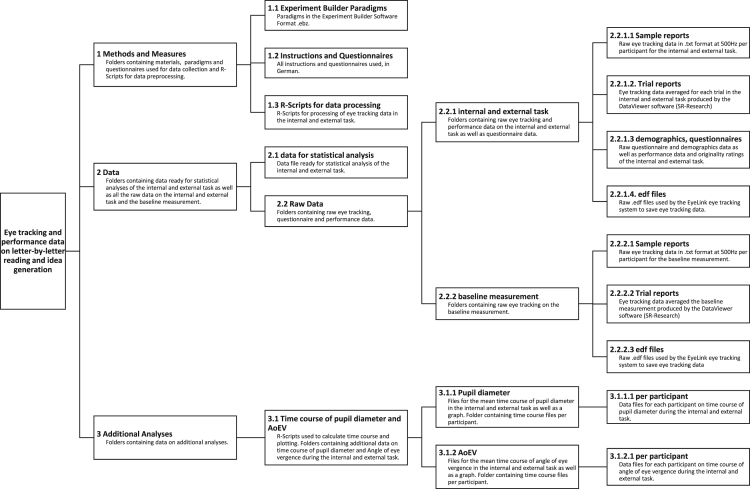
Folder structure of provided data in the Open Science Framework repository (https://osf.io/fh66g/[5]).

Data [5] provided at the Open Science Framework (OSF) include demographic information for each participant, eye tracking data, task performance measures, questionnaire data, R-scripts, instructions and visualizations of additional analyses. At OSF, data [5] are organized in three main components (see Fig. 1): ‘1 Methods and Measures’, ‘2 Data’ and ‘3 Additional Data’.

In the component ‘1 Methods and Measures’, you find the eye tracking paradigms in the subcomponent ‘1.1 Experiment Builder’. We wrote the eye tracking paradigms with the manufacturers Software Experiment Builder [7] and can only opened with this software.

**Fig. 2:**
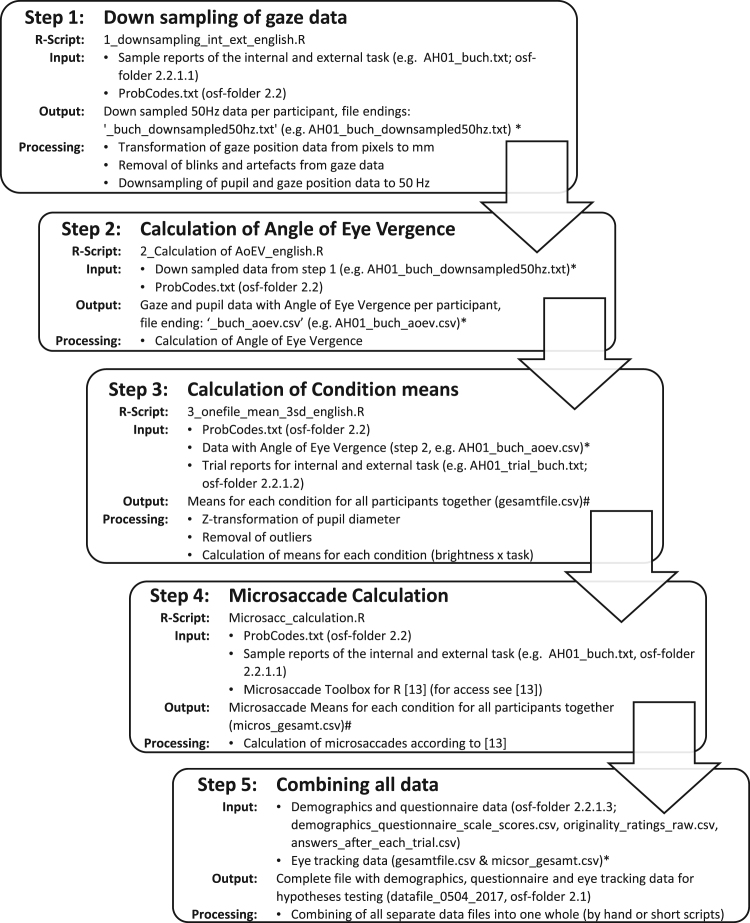
Preprocessing steps of the eye tracking data of the creativity and letter-by-letter reading task. * Data not provided because those files can be reproduced with available data and only serve to save data between analyses steps. # not provided because data are already included in the final file ‘datafile_0504_2017.csv’. osf-folder=folder number within the Open Science Framework repository (https://osf.io/fh66g/[5]).

Instructions of each task and questionnaires are hosted in the subsection ‘1.2 Instructions and questionnaires’. The R-scripts used to analyze eye-tracking data of the idea generation and letter-by-letter reading task are in the subsection ‘1.3 R-scripts for data analyses’. Fig. 2 provides information on how to use those R-scripts.

In the component ‘2 Data’ you find the complete data set used for all analyses and the raw data in the subsection ‘2.2 Raw Data’. ‘2.2 Raw Data’ has separate sections for the tasks (‘2.2.1 internal and external task’, ‘2.2.2 baseline measurement’). Eye tracking raw data, demographic data and responses to questionnaires are within those subsections. Sections ‘2.2.1.1 and 2.2.2.1 Sample reports’ comprise 500 Hz raw data in text format for each participant on gaze position, pupil diameter and events. Sections ‘2.2.1.2 and 2.2.2.2 Trial reports’ host raw trial-wise statistics on saccades, blinks and fixations. Sample reports and Trial reports were generated with the DataViewer Software [11]. The edf-files (special file format used by the Software of SR-Research) are in the sections 2.2.1.4 and 2.2.2.3. Those edf-files are readably with Software from SR-Research or special scripts and are required if one analyses the data with different thresholds for blink, fixation or saccade detection.

**Fig. 3:**
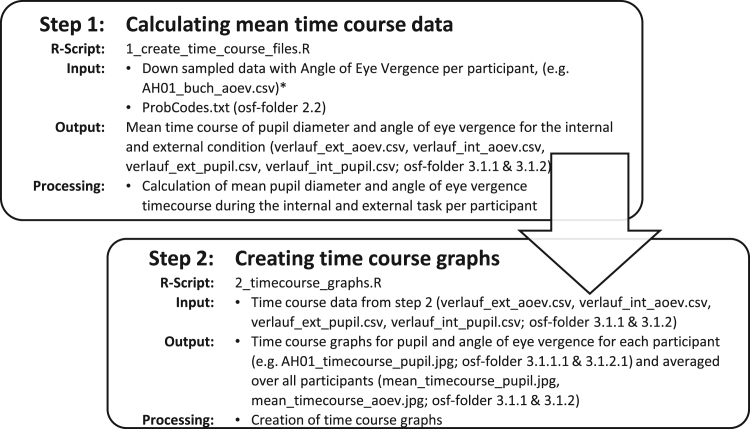
Steps to create the time course data and graphs of pupil diameter and angle of eye vergence during the creativity and letter-by-letter task. * Data not provided, see Fig. 1 step 2. osf-folder=folder number within the Open Science Framework repository (https://osf.io/fh66g/[5]).

Supplementary analyses on the time course of pupil diameter and angle of eye vergence with graphs are in the component ‘3 Additional Analyses’. Fig. 3 explains the steps to create the hosted time course data and graphs.

## 2 Experimental design, materials and methods

Participants were 50 young adults (23±4 years old, 32 females, 17 males, 1 non-binary gender identity), mainly university students from Graz Austria. Participants received course credits and/or participated in a raffle to win a skiing holiday. None of the participants reported strabismus or other medical conditions affecting vision and had normal or corrected-to-normal (soft contact lenses) vision. All participants gave written informed consent and the study was approved by the local ethics committee. Further details on demographics can be found in the subcomponent ‘2.2.1.3 demographics and questionnaires’ in the data repository [5].

### 2.1 Reading and idea generation task

Instructions for the reading and idea generation task can be found in the subcomponent ‘1.2 Instructions and questionnaires’ and the paradigms in the subcomponent ‘1.1 Experiment Builder’ of the data repository [5].

In the letter-by-letter reading and the idea generation task, a stream of letters was presented in the center of the screen. All letters were lowercase, and presented in black Arial font of size 15 pt resulting in a character width of 0.32° visual angle (ca. 2.5 mm or 8.5 pixels). Umlauts were written-out (e.g. “ä” to “a–e”) and words were separated by a dash (“-“) instead of a space so the screen was never empty. Letters were presented for 500 ms each, followed by the next letter. Background luminance was brighter (RGB color code: 204,204,204) in half of the trials and darker (102,102,102) in the other half.

Participants performed eight experimental trials of a reading task and eight trials of an idea generation task preceded by one practice trial of each task. The experimental trials were organized in two blocks with a break in between. Each block comprised four consecutive reading trials and four consecutive idea generation trials (half with dark and half with bright background, respectively). Half of the participants started with the reading task, the other half with the idea generation task. After each block, participants filled out a short questionnaire regarding aspects of task performance, including questions about their concentration, the perceived demands of tasks and the amount of distraction by the letter stream. Answers to those questions can be found in the subcomponent ‘2.2.1.3 demographics, questionnaires’ [5]. Procedure is illustrated in Fig. 1 in Ref [8].

#### 2.1.1 Letter-by-letter reading task

Participants read a German text of 120-character length. Text was presented as a stream of consecutive letters, each presented for 500 ms at the middle of the screen, resulting in presentation duration of 60 s for the entire text. After text reading, two comprehension questions on the content of the message were presented on the screen (e.g. text: “Albert and I are going to a concert on Tuesday. I asked Franz if he wants to join us, but he visits his grandmother that day” Questions: “What day are we going to the concert?”, “Who is Franz visiting?”). In a third question, participants had to indicate the direction of attentional focus during the task, from 0=“totally absorbed in thoughts” to 5=“totally focused on external events”. Participants answered the questions aloud. Answers can be found in the subcomponent ‘2.2.1.3 demographics and questionnaires’ [5]. At the end of each trial participants pressed the spacebar to continue with the next trial.

#### 2.1.2 Idea generation task

We used the alternate uses task [1]. The alternate uses task asks to generate creative uses for a common household object. The following objects were used: paper cup, tinfoil, picture frame, paperclip, plastic bottle, CD, elastic band and match box; additionally, credit card was used as object for the practice trial.

The procedure of the idea generation task was essentially the same as in the reading task: at the beginning of each trial participants received the instruction. Then they had 60 s to think of creative uses without verbalizing them. During this period, the same letters as in the reading task were presented one character at a time, but in reverse order to make it unreadable. At end of each task, participants were prompted to tell their two best ideas. Finally, participants had to indicate again their attentional focus during the task. Creativity of the generated ideas was rated by four experienced raters on a four-point scale ranging from 0=“not creative” to 3 “very creative” [9]. Answers and originality ratings of the ideas can be found in the subcomponent ‘2.2.1.3 demographics and questionnaires’ [5].

### 2.2 Baseline measurement

Instructions for the baseline measurement can be found in the subcomponent ‘1.2 Instructions and questionnaires’ and the paradigm in the component ‘1.1 Experiment Builder’ [5]. Participants were asked to hold their gaze on a fixation cross for 60 s. Fixation cross was a black plus sign in Arial, size 15 pt in the center of the screen, background was RGB color code: 204,204,204.

### 2.3 Questionnaires

Between blocks participants filled out personality questionnaires in the antechamber. Questionnaires were presented using LimeSurvey [10], paper versions of the questionnaires can be found at OSF in the subcomponent ‘1.2 Instructions and questionnaires’ [5]. The short imaginal processes inventory (SIPI, [2]) and the daydreaming frequency scale (DDFS, [3]) were translated to German. The German version of the Runco ideational behavior scale (RIBS, [4]) was presented. Raw answers to those questionnaires can be found at OSF in the subcomponent ‘2.2.1.3 demographics, questionnaires’ [5] and the scale scores are included in the overall data file ‘Data’ [5].

### 2.4 Eye tracking

Eye data were recorded during the performance periods (60 s) of the letter-by-letter reading task, the idea generation task and during the 60 s baseline measurement. Binocular eye data were recorded using an EyeLink 1000 Plus Tower Mount eye tracker (SR Research, Ontario, Canada) with a temporal resolution set to 500 Hz. For stimulus presentation and response recording, the Experiment Builder software [7] was used. For calibration, validation, drift correction and computation of the eye movement parameters (blinks, fixations, saccades), we used the manufacturer's software [7], [11]. A velocity threshold for saccade detection was 35°/s and acceleration threshold was 9500°/s^2^. A 9-point calibration procedure before each block and a drift correction before each trial was performed. Spatial resolution was typically better than 0.30°.

Participants sat in a sound attenuated room with the lights on and at a distance of 50 cm from the screen. Their heads were stabilized using chin rest and forehead rest of the EyeLink Tower Mount (SR Research, Ontario, Canada). Stimuli were presented on a 19-in LG flatroon L1920P monitor run at 60 Hz and a 1240×1024 pixels resolution, subtending 29.4 pixels per degree visual angle. Participants’ answers were recorded with a microphone to monitor and record task performance.

#### 2.4.1 Analyses of eye tracking data

At OSF, in the component ‘2.2 Raw Data’ you can find the raw.edf files (2.2.1.4 and 2.2.2.3 edf files) as outputted by the eye tracker and the.txt files (2.2.1.1 and 2.2.2.1 Sample reports; 2.2.1.2 and 2.2.2.2 Trial reports) as outputted by the Data Viewer [11]. Fixation durations and counts of fixations, blinks and saccades and saccade amplitude per trial were calculated using Data Viewer [11]. Saccades were defined as eye movements that exceeded 30°/s velocity, 8000°/s^2^ acceleration and/or 0.15° motion. Blinks were defined as a period with the pupil data missing for three or more samples in a sequence (=at least 6 ms) and fixations were defined as any period that is not a blink or a saccade.

Further analyses were performed in R [6] and steps of processing and needed files are described in Fig. 2. R-scripts for gaze data analyses are in the OSF component ‘1.3 R-scripts for data processing’ [5]. Only data for which the eye tracker had recorded both eyes were analyzed. Blinks as well as additional 200 ms periods before and after each blink were removed from gaze position and pupil diameter data. For calculation of pupil diameter and eye vergence, eye tracking data were down-sampled from 500 Hz to 50 Hz by averaging across 10 data points (20 ms). Pupil diameter data were transformed from arbitrary units (measured by the eye tracking software) to *z*-values to make them comparable across subjects.

Calculation of angle of eye vergence was similar to methods applied in previous research [12].

For microsaccade calculation, original 500 Hz gaze position data were used. Microsaccades (count and amplitude) were determined using the Microsaccade Toolbox for R [13] with *λ*=4 and a minimum microsaccade duration of 6 ms. Microsaccades were defined as saccades with an amplitude smaller than 1.0° [14]. Only binocular microsaccades with a minimum overlap of one data sample were considered. Microsaccade amplitude was defined as the mean across both eyes.

For all eye parameters, means or within-trial variability was computed for each trial. Trials with more than 50% missing data and trials with values beyond three standard deviations from the individuals mean were discarded. Remaining trials were averaged for the reading and the idea generation trials and for the bright and darker background luminance, respectively.

#### 2.4.2 Additional analyses of time course of pupil diameter and angle of eye vergence

Calculation (R-scripts) and visualization of 60 second time course of pupil diameter and angle of eye vergence averaged per task (reading task and idea generation task) can be found at OSF in the component ‘3 Additional analyses’ [5]. Tables and graphs of time course for each participant and the averaged time course over all participants are provided in subsections of this folder. Fig. 3 explains the creation of the time course tables and graphs.
